# A Study on the Hall–Petch Relationship and Grain Growth Kinetics in FCC-Structured High/Medium Entropy Alloys

**DOI:** 10.3390/e21030297

**Published:** 2019-03-19

**Authors:** Yung-Chien Huang, Che-Hsuan Su, Shyi-Kaan Wu, Chieh Lin

**Affiliations:** 1 Department of Materials Science and Engineering, National Taiwan University, Taipei 106, Taiwan; 2 Department of Mechanical Engineering, National Taiwan University, Taipei 106, Taiwan

**Keywords:** high entropy alloy, Hall–Petch relationship, grain growth, activation energy

## Abstract

The recrystallization behavior, grain growth kinetics, and corresponding hardness variation of homogenized and 80% cold-rolled FeCoNiCrPd, FeCoNiCrMn, and their quaternary/ternary FCC-structured high/medium entropy alloys (H/MEAs) annealed under different conditions were investigated. Experimental results indicate that the grain size and hardness of these H/MEAs follow the Hall–Petch equation, with the Hall–Petch coefficient *K*_H_ value being mainly dominated by the alloy’s stacking fault energy and shear modulus. The FeCoNiCrPd alloy exhibits the highest hardness of the H/MEAs at the same grain size due to the largest Young’s modulus difference between Cr and Pd. The grain growth exponent *n*, kinetic constant *k*, and activation energy for grain growth *Q_G_* of all H/MEAs are calculated. The *k* can be expressed by the Arrhenius equation with *Q_G_*, which is attributed to the diffusion rate. The results demonstrate that the *Q_G_* values of these H/MEAs are much higher than those of conventional alloys; most notable is FeCoNiCrPd HEA, which has an unusually lattice distortion effect that hinders grain growth.

## 1. Introduction

High entropy alloys (HEAs) are attracting attention for their promising mechanical properties and intriguing concepts in alloy design [1,2,3,4,5,6,7,8]. Yeh et al. hypothesized that HEA contained at least five principal elements with equiatomic or near-equiatomic compositions, which would result in high configurational entropy of this multi-principal-element alloy (MPEA) [1]. The high configurational entropy would favor the formation of solid solutions rather than intermetallics by overwhelming the enthalpy of compound formation; i.e., HEAs would develop a single-phase alloy without precipitation. A famous example is the equiatomic FeCoNiCrMn HEA with a FCC-structured single phase, which was first reported by Cantor et al. [2]. The subsequent studies on FeCoNiCrMn HEA indicated its high degree of phase stability under various preparation processes and thermomechanical conditions, and its excellent mechanical properties at ambient or cryogenic temperatures [9,10,11,12,13,14,15,16,17,18]. However, recent studies have pointed out that secondary phases or precipitates appear in most HEAs [19,20,21,22]. It seems that the initial concept of high configurational entropy is unlikely to overcome the phase separation. Therefore, the terms including MPEAs and complex concentrated alloys (CCAs) are introduced to comprise all alloys containing at least five principal elements with equiatomic or near-equiatomic compositions regardless of whether they are single-phase or multi-phase, while the term HEAs is intended for single-phase MPEAs [4].

It is well known that grain refinement is an effective way to improve the mechanical properties with a combination of high strength and good ductility. Previous studies have shown that the grain size of HEAs can be controlled by rolling and recrystallization [9,12,13,23]. The ternary/quaternary derivatives of the FeCoNiCrMn HEA with no Mn included have also been systematically studied to determine their phase stabilities, microstructure, and microhardness evolution [22]. However, the factors that affect the grain growth behavior of HEAs and the corresponding activation energies of grain growth kinetics have not been fully clarified, although they are crucial for designing alloys for future engineering applications. In this study, the grain growth behavior and Hall–Petch relationship exhibited by FeCoNiCrPd HEA, FeCoNiCrMn HEA, and their ternary/quaternary derivatives with the Mn included were studied and compared with the traditional alloys. The factors affecting the grain growth characteristics and the grain refinement hardening revealed in these high/medium entropy alloys (H/MEAs) are also discussed.

## 2. Materials and Methods 

Ingots of equiatomic FeCoNiCrMn, FeCoNiCrPd, FeCoNiCr, FeCoNiMn, CoNiCr, and CoNiMn H/MEAs were fabricated in a vacuum arc remelter (VAR), and the purity of each constituent element was higher than 99.9 wt %. A pure titanium button was used as the getter to absorb the residual oxygen before the melting. Due to the high vapor pressure of Mn, 50–50 wt % Ni-Mn master alloy with purity >99.9 wt % was purchased from Testbourne Ltd., Hampshire, England, UK, and used to prepare the Mn-containing H/MEAs to minimize the weight loss of Mn. The ingots were flipped and remelted six times under a high purity argon atmosphere to ensure their chemical homogeneity. The ingots were then homogenized in the tube furnace with the argon flow for 24 h at as high a temperature as possible according to their melting points to provide sufficient diffusion energy and prevent melting. The CoNiCr, FeCoNiCr, FeCoNiCrMn, and FeCoNiCrPd ingots were homogenized at 1200 °C, while the CoNiMn and FeCoNiMn ingots were homogenized at 1100 °C. The melting points and the corresponding homogenization temperatures of these H/MEAs are reported in the previous studies [22,24,25]. The structure of these H/MEAs after homogenization are also presented in the previous studies and all H/MEAs show the FCC structure [22,26]. The homogenized ingots were rolled into plates at room temperature with a thickness reduction of 80%. The specimens were then cut from the plates to appropriate sizes, heated under different annealing conditions, and quenched in water. 

For the investigation of the grain growth kinetics of H/MEAs, the annealing treatments of the specimens were conducted at temperatures of 800 °C to 1100 °C /1200 °C, based on the alloy’s melting point, and for annealing times of 1 h to 4 h. The annealed specimens were ground, polished and etched in the solution of H_2_O:HCl:CuSO_4_ = 50 mL:50 mL:10 g, and then observed by optical microscopy. Thereafter, the grain sizes of the annealed specimens were determined from the averages of 10 measurements performed by the linear intercept method according to ASTM E112-12 [27]. At the same time, each specimen’s hardness was measured by a Vickers microhardness tester (Mitutoyo HM, Japan) with an applied load of 500 gf and a loading time of 15 s. The hardness value of each specimen was averaged from 10 tests, with the largest and the smallest values excluded. The average diagonal length of Vickers microhardness is ~75 μm for ~200 Hv when the applied load is 500 gf. The indentation can sample several grains for the alloys with small grain size. Although the grain size of the H/MEAs annealed at higher temperatures is larger than the size of indentation, which means that the hardness might be measured from only one or two grains and might be affected by the grain orientation, the average procedure from 10 sampling positions to determine the hardness can reduce the grain orientation effect.

## 3. Results and Discussion

**Figure 1:**
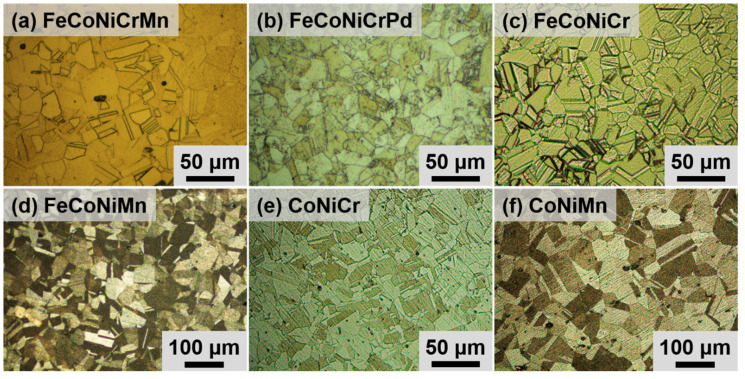
Optic images of (**a**) FeCoNiCrMn, (**b**) FeCoNiCrPd, (**c**) FeCoNiCr, (**d**) FeCoNiMn, (**e**) CoNiCr, (**f**) CoNiMn alloys annealed at 900 °C for 1 h.

**Figure 2:**
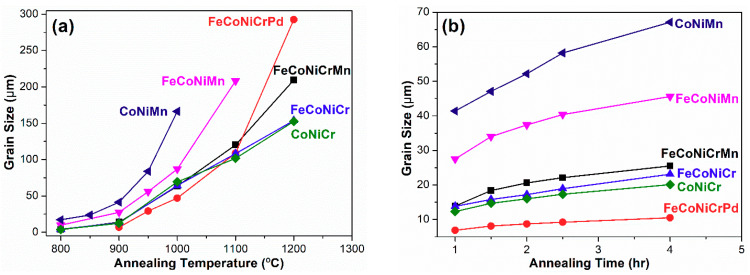
(**a**) Grain size evolution of all high/medium entropy alloys annealed at different temperatures for 1 h. (**b**) Grain size evolution of all high/medium entropy alloys annealed at 900 °C for different times.

Figure 1 shows representative recrystallized microstructures of all the 80% cold-rolled alloys annealed at 900 °C for 1 h. All alloys possess equiaxed grains with the FCC structure after various annealing treatments [22,26,28]. Annealing twins appear in some grains, and the grain having the annealing twin is regarded as a single grain during the grain size measurement [27]. The effect of grain refinement on hardness is evaluated by Vickers microhardness tests. The grain sizes and Vickers microhardnesses of all alloys annealed at all temperatures for 1 h are listed in Table 1. Experimental results on the evolution of the grain size for all alloys annealed at all temperatures for 1 hr are plotted in Figure 2a, and those of alloys annealed at 900 °C for all annealing times are shown in Figure 2b.

### 3.1. Grain Size Effect on Microhardness

**Figure 3:**
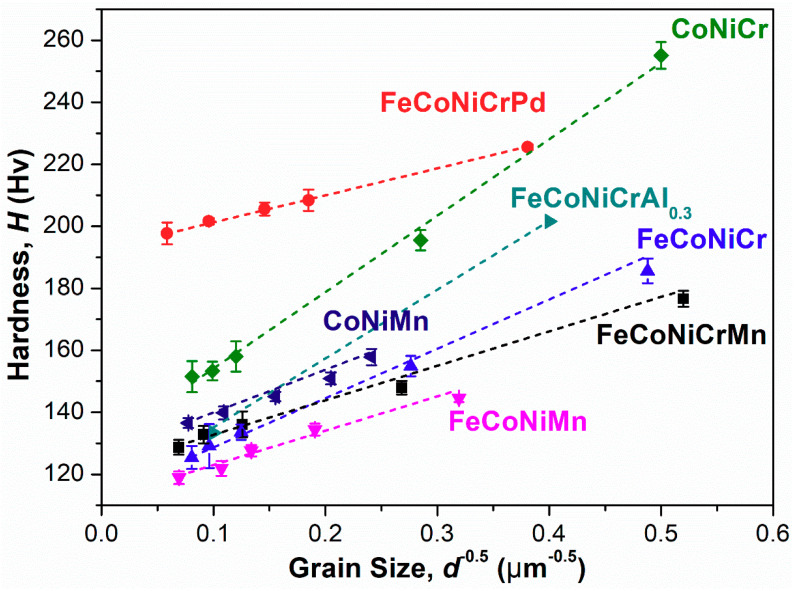
Hardness *H* as a function of grain size *d* for all high/medium entropy alloys annealed at different temperatures for 1 h to study the Hall–Petch relationship.

Figure 3 plots the curves of hardness *H* vs. grain size *d* for recrystallized specimens of all alloys for all annealing conditions. The data of the FeCoNiCrAl_0.3_ FCC-structured alloy reported by Gwalani et al. are also included for comparison [23]. From Figure 3, one can find that the hardness *H* of the annealed specimen decreases as its grain size *d* increases. These data follow the Hall–Petch relationship, as expressed by Equation (1) [29]

(1) $$H=H_{0}+K_{H}d^{-1/2}$$

where *H*_0_ is the intrinsic hardness of the alloy, *d* is the average grain size, and the *K*_H_ value is the Hall–Petch coefficient, which can be determined from the slope of the Hall–Petch curve shown in Figure 3. Table 2 lists the *H*_0_ and *K*_H_ values of all alloys and that of FeCoNiCrAl_0.3_ alloy. It has been proposed that the factors of lattice distortion (δ), stacking fault energy (SFE) and shear modulus exhibited in H/MEAs can hinder the movement of the dislocations and affect the *K*_H_ value [30]. The severely distorted lattice in HEAs would make the dislocation line not straight which leads to more difficulty in the dislocation motion [12]. Here, the δ value is calculated from Equation (2) [31] and is also shown in Table 2.

(2) $$\delta =\sqrt{\sum_{i=1}^{n}c_{i}{\left(1-r_{i}/\bar{r}\right)}^{2}},\;\bar{r}=\sum_{i=1}^{n}c_{i}r_{i}$$

where *c_i_* and *r_i_* represent the atomic percentage and the atomic radius of the *i*th element, respectively, and *n* is the number of alloying elements. According to Table 2, the extent of the δ value exhibited in H/MEAs increases in the following order: FeCoNiMn < CoNiMn < FeCoNiCrMn < FeCoNiCr < CoNiCr < FeCoNiCrAl_0.3_ < FeCoNiCrPd, which is hardly correlated to the order of the *K*_H_ values. The FeCoNiCrPd alloy with the largest δ value exhibits the smallest *K*_H_ value, while the CoNiCr alloy possesses the third largest δ value and has the largest *K*_H_ value at the same time, indicating that the δ value exhibited in H/MEAs does not affect the *K*_H_ value. On the other hand, in general, the *K*_H_ value of the conventional alloy is small when the alloy has a high SFE and a low shear modulus simultaneously [22,30]. Fischmeister and Karlsson also noted that the *K*_H_ value decreases with increases in SFE [32]. Since the cell structure forms predominantly in high SFE alloys, the cell boundaries would restrict the slip lengths of dislocations [32,33]. It has been reported that the addition of metallic elements in Ni can reduce the SFE of the Ni with the effect in the following order: Cr > Al > Co > Fe > Mn > Pd [34,35,36,37]. Therefore, as calculated from the total effect of the SFE reduction weighted by the contents of the different constituent elements, the SFE order of these alloys would be FeCoNiCrPd > FeCoNiMn > CoNiMn > FeCoNiCrMn (30) [38] > FeCoNiCr (27) [36] > FeCoNiCrAl_0.3_ (<30) [39,40] > CoNiCr (18–22) [36,41]. The numbers in round brackets are corresponding SFE values for H/MEAs which have been measured in the previous studies. Although it is certainly more complex when considering the interactions among all elements rather than the only effect of SFE reduction by adding the elements in Ni, the consistency between the orders of the measured and expected values demonstrates the validity of the SFE reduction estimation. The expectation of the SFE order exhibited in H/MEAs almost agrees with the order of the *K*_H_ values listed in Table 2, except for the FeCoNiCrMn alloy, suggesting there may be another factor that affects the *K*_H_ value. From Table 1, one can find that the deviation of the grain size in the FeCoNiCrMn alloy is the highest among all alloys under the same *d*. This feature may have caused the misfit in the order of the *K*_H_ values exhibited in FeCoNiCrMn HEA and will require further study.

In addition to the SFE, the reported literature has also demonstrated that the alloy’s shear modulus will change the *K*_H_ value [30]. It is well known that the dislocation motion is greatly related to the shear stress. Slip occurs when the shear stress on the slip plane and in the slip direction reaches the critical resolved shear stress. Therefore, the shear modulus is a critical parameter in many models of the dislocation behaviors including the approximation of *K*_H_ value [33,42]. Table 2 also shows the shear moduli of all alloys but the FeCoNiCrPd alloy [18]. From Table 2, one can see that the CoNiCr alloy has the largest shear modulus of 87 GPa, while those of the FeCoNiCr and FeCoNiCrMn alloys are 84 GPa and 80 GPa, respectively. The alloys with Cr replaced by Mn,—i.e., FeCoNiMn and CoNiMn—possess the lowest shear modulus of 77 GPa. Therefore, the substitution of Cr for Mn increases the shear modulus and results in the *K*_H_ values of CoNiCr and FeCoNiCr being higher than those of CoNiMn and FeCoNiMn, respectively. Obviously, the above discussion demonstrates that the variation of the *K*_H_ value exhibited in H/MEAs is mainly dominated by the combination of the factors of the alloy’s SFE and shear modulus. For the comparison with the results in the previous study, the Hall–Petch values of the FeCoNiCrMn HEA measured by Liu et al. [12] are listed in Table 2. Note that the *K*_H_ value of FeCoNiCrMn HEA by Liu et al. is lower than that in this work. The discrepancy between the *K*_H_ values might result from the difference in the pre-treatments and is discussed later.

### 3.2. Solid-Solution Effect on Microhardness

According to Figure 3, under the same *d*, the CoNiCr and CoNiMn ternary alloys are harder than the FeCoNiCr and FeCoNiMn quaternary alloys. Wu et al. [22] also found similar results on other alloy systems and pointed out that the nature of the alloying elements needs to be considered. Factors that influence the alloy’s solid-solution strengthening would include the differences in the atomic size and the Young’s modulus of the alloying elements. When the differences in both the atomic size and the Young’s modulus increase, the extent of the δ value increases simultaneously, which would impede the movement of the dislocations [43]. Table 3 lists the atomic sizes and the Young’s moduli for the alloying elements [44,45,46]. To be more representative in HEAs, the effective atomic radius of each element in FeCoNiCrMn HEA calculated by the “Effective Atomic Radii for Strength” (EARS) methodology is listed in Table 3 [47]. In addition, due to almost the same value as the effective atomic radius, the metallic radii are also listed as the reference to show the atomic radii for Pd and Al. From Table 3, for all alloys, the largest atomic size difference among these alloying elements is that between Ni and Pd, which is only 10.4%, while the largest modulus difference—that between Cr and Pd—is 130.5%. Therefore, according to the measured alloy’s hardness, it is likely that the hardness (strengthening) variation is greatly affected by Young’s modulus misfit rather than by atomic size misfit. In the present study, when the Mn element of CoNiMn and FeCoNiMn alloys is replaced by Cr to yield CoNiCr and FeCoNiCr alloys, respectively, the Young’s modulus difference increases due to the prominently high modulus of Cr, thereby increasing the hardness. Table 3 also lists the Young’s modulus of Al being only 70 GPa, suggesting that the addition of Al into the alloy would increase the modulus misfit and hence the hardness. Figure 3 shows great agreement with the estimation by comparing FeCoNiCr alloy and FeCoNiCrAl_0.3_ alloy; the latter is harder than the former. In addition, although the Young’s modulus of Al is smaller than that of Pd, the content of Al in FeCoNiCrAl_0.3_ alloy is only three tenths that of the other constituent elements, which gives rise to less of an effect on the Young’s modulus difference than that between Cr and Pd in FeCoNiCrPd alloy. Therefore, FeCoNiCrPd alloy can be expected to exhibit the highest hardness among these alloys at a given grain size, which also matches the results shown in Figure 3.

### 3.3. Grain Growth Kinetics Analysis

#### 3.3.1. Grain Growth Exponent 

The kinetics of grain growth for recrystallized specimens is deduced by analyzing the grain size as a function of time, as shown in Figure 2b, in accordance with the classical kinetic theory for grain growth [48,49]

(3) $$d^{1/n}-d_{0}^{1/n}=kt$$

where *d* is the mean grain size of recrystallized alloys annealed at temperature *T* for time *t*, *d*_0_ is the initial mean grain size before annealing, *n* is the grain growth exponent, and *k* is the kinetic constant, which is dependent on the temperature *T*. Usually, *d*_0_ is much smaller than *d* in specimens heavily cold-rolled and then annealed at high temperature, due to the high storage strain energy driving the rapid grain growth. Thus, the Equation (3) can be simplified as [50]

(4) $$d^{1/n}=kt$$

**Figure 4:**
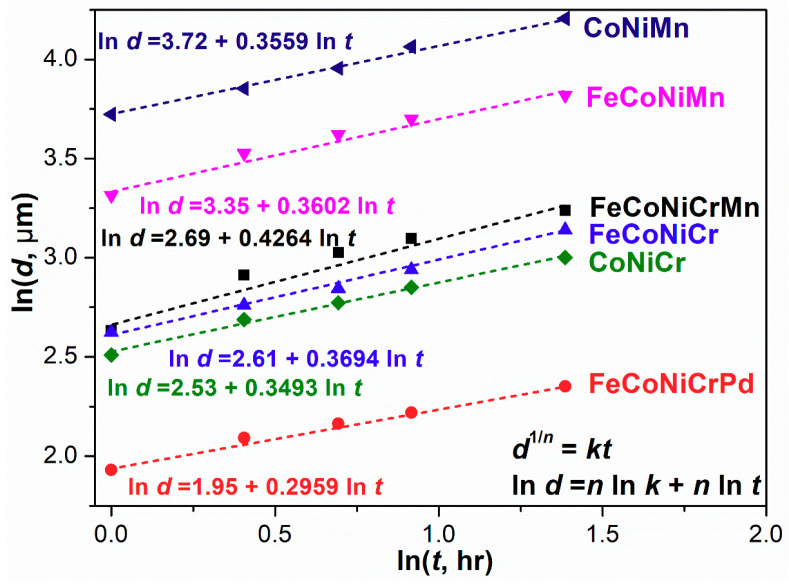
Grain size as a function of annealing time at 900 °C for all high/medium entropy alloys.

The grain growth exponent *n* and the kinetic constant *k* can be figured out from the plot of the logarithm of both grain size *d* and time *t* shown in Figure 4. From Figure 4, the *n* and *k* values for all alloys are measured, and the data are listed in Table 4. 

In a theoretical case, the *n* value of pure metal is 0.5 [51]. However, in most experimental studies on alloys, the *n* values have been reported to be less than 0.5 due to the solute drag effects, which lower the grain boundary mobility [49,52,53,54]. This effect can be quantified by the solute drag factor, which is proportional to the solute concentration and inversely proportional to the grain boundary mobility [55]. However, for equiatomic H/MEAs, the determination of the solute element is difficult due to the absence of a sole principal element. The solute drag effect would be much more complicated and needs further investigations. 

#### 3.3.2. Activation Energy Q_G_ for Grain Growth

**Figure 5:**
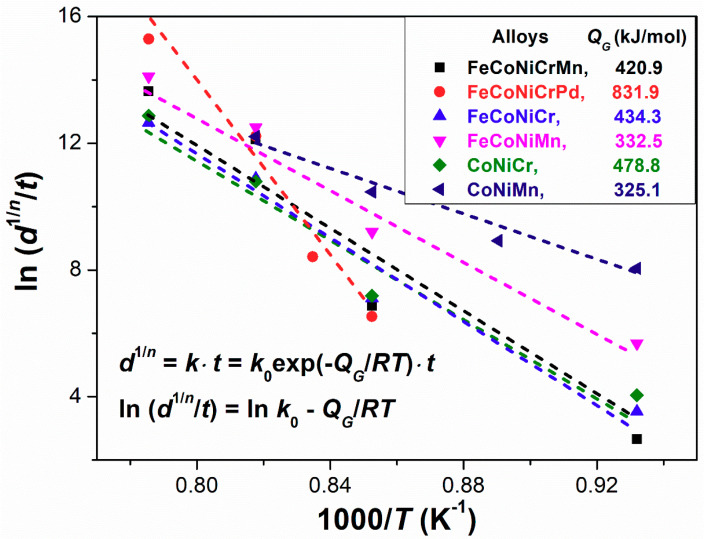
Grain growth kinetic constant *k* (*d*^1/*n*^/*t*) as a function of the reciprocal of absolute temperature 1000/*T* for all high/medium entropy alloys.

The grain growth kinetic constant *k* of Equation (4) can be expressed in an Arrhenius form which depends on the temperature *T*, as shown by Equation (5) [51]

(5) $$k=k_{0}\exp\left(-Q_{G}/RT\right)$$

where *k*_0_ is a pre-exponential constant, *R* is the gas constant, and *Q_G_* is the activation energy for grain growth. To obtain the *Q_G_* value, Equation (5) can be adapted by using Equation (4) in logarithm form to yield Equation (6), and then the ln (*d*^1/*n*^/*t*) vs. 1/*RT* profile can be plotted, as shown in Figure 5.

(6) $$\ln\left(d^{1/n}/t\right)=\ln k=\ln k_{0}-\left(Q_{G}/RT\right)$$

From Figure 5, the *Q_G_* values of all alloys are measured, and their data are listed in Table 5. The *Q_G_* values of some conventional alloys and a HEA investigated by Liu et al. are also included in Table 5 for comparison [12,56,57,58]. Based on Equations (6) and (4), it is demonstrated that, for the case of the larger *Q_G_* value, a certain amount of temperature change (fixed Δ*T*) will lead to higher variation in the grain growth constant (large Δ*k*), thereby increasing the difference in the grain sizes (large Δ*d*) at two given temperatures at a fixed time interval (fixed Δ*t*). 

Generally, the proposed factors that affect the grain growth kinetic *k* are the drag effect by precipitates, diffusion rate, texture, and heterogeneities exhibited in an alloy [61]. In the present study, all alloys possess the FCC-structured single phase and show no precipitates [22]. All H/MEAs used in this study were homogenized. The recrystallized microstructures of all alloys have also been observed to have only rare differences in texture [22,28]. Therefore, the discrepancy in the *Q_G_* value among these alloys may be attributed to the diffusion effect. The data of the diffusion coefficients *D* and other related diffusion parameters of these alloys are listed in Table 5 [25,59,60], in which *D* can also follow the Arrhenius equation shown in Equation (7) [62]

(7) $$D=D_{0}\exp\left(-Q_{D}/RT\right)$$

where *R* is the gas constant, *Q_D_* is the activation energy for diffusion, and *D*_0_ is the intrinsic diffusion constant. According to Table 5, the *Q_G_* values of different alloys decrease with increases in the *D* value and show little relation to the *Q_D_* values. It is reasonable that the high *D* value can enhance the grain growth, which is a diffusion-dependent process for the movement of grain boundaries, thereby causing the low *Q_G_* value. Previous studies have shown that the element Mn in FeCoNiCrMn HEA has the highest diffusion rate among all the constituent elements [59,60]. Therefore, according to Table 5, the substitutions of Mn for Cr in CoNiCr and FeCoNiCr alloys can lower the *Q_G_* values; i.e., CoNiCr (478.8 kJ/mol) → CoNiMn (325.1 kJ/mol); FeCoNiCr (434.3 kJ/mol) → FeCoNiMn (332.5 kJ/mol). In addition, the addition of Mn into FeCoNiCr alloy can also decrease the *Q_G_* value; i.e., FeCoNiCr (434.3 kJ/mol) → FeCoNiCrMn (420.9 kJ/mol). These results reveal the important role of Mn in affecting the *Q_G_* value by increasing the diffusion rate of alloys. Note that the lattice distortion δ is also related to the diffusion coefficient shown in Table 5; it can be presumed that the diffusion would decelerate due to the lattice distortion hindering the movement of the atoms. 

Compared to the conventional alloys shown in Table 5, such as stainless steel [57], magnesium alloys [58,63], and titanium alloy [56], the *Q_G_* values of all alloys in this study are much higher. In addition, as shown in Table 5, Liu et al. [12] measured the *Q_G_* value of FeCoNiCrMn HEA to be 321.7 kJ/mol, which is lower than the *Q_G_* value of 420.9 kJ/mol found in this study. Besides, according to Table 2, the *K*_H_ value of FeCoNiCrMn HEA acquired by Liu et al. is also lower than that in this study. It is supposed that the differences in the *Q_G_* values and the *K*_H_ values of these two FeCoNiCrMn HEAs may have resulted from the different pre-treatments before recrystallization. The FeCoNiCrMn HEA from Liu et al. was remelted four times, followed by 70% cold-rolling without homogenization, while the alloy in this study was remelted six times, homogenized at 1200 °C, and then 80% cold-rolled. According to the basic theory of recrystallization, the driving force for recrystallization accounts for the release of the stored strain energy by cold-rolling. The recrystallization rate increases with increasing amount of cold-rolling and recrystallization temperature. Compared to Liu’s work, the alloy in this study was cold-rolled with more reduction in thickness and further annealed at higher temperatures of 800–1200 °C while the alloy was annealed at 850–950 °C in Liu’s study. Therefore, the incubation period for recrystallization before steady grain growth would be shorter in this study, leading to a longer time for grain growth in a finite annealing time. For the alloys annealed at 900 °C for 1 h in two studies, it is revealed that the grain size of FeCoNiCrMn alloy in this study (13.9 μm) is larger than that in Liu’s study (6.9 μm). Based on the grain growth theory, the driving force for grain growth lies in the surface energy of grain boundaries. Since the grain size in this study is larger under the same annealing condition due to the pre-treatment, the grain boundary area diminishes and the total surface energy decreases. The less driving force for grain growth would decelerate the grain boundary migration and hence increase the *Q_G_* value. In addition, the non-homogenized alloy would cause not only the reduced lattice distortion due to the less random mixing of elements but also the less SFE reduction due to the less alloying of other elements in Ni. Therefore, based on the mechanisms of the lattice distortion and the SFE affecting the *K*_H_ value mentioned in Section 3.1, the difference of the *K*_H_ value between the same alloys in two study may be caused by the composition variation. It is suggested that the causes of the differences in the *Q_G_* values of the same HEAs fabricated for the equiatomic composition may be deviation in the alloy’s composition from the equiatomic ratio, the impurities contained in raw materials, the annealing temperatures, and the different cold-rolling amounts before recrystallization annealing.

## 4. Conclusions

To study the Hall–Petch relationship and the grain growth kinetics, homogenized and 80% cold-rolled FeCoNiCrPd, FeCoNiCrMn, and their quaternary (FeCoNiCr, FeCoNiMn) and ternary (CoNiCr, CoNiMn) FCC-structured H/MEAs were heated at various annealing temperatures and for various times. Experimental results indicate that, after annealing at 800–1200 °C for 1 h, all 80% cold-rolled and recrystallized H/MEAs follow the Hall–Petch equation, $H=H_{0}+K_{H}d^{-1/2}$. The stacking fault energy and the shear modulus exhibited in these H/MEAs are the major factors that affect the *K*_H_ value. The addition of Mn or the substitution of Mn for Cr in H/MEAs can lower the alloy’s shear modulus, thereby decreasing the alloy’s *K*_H_ value. At a given grain size, the hardness of these alloys is greatly affected by the Young’s modulus difference among the constituent elements. The FeCoNiCrPd alloy exhibits high hardness due to the large modulus difference between Cr and Pd. The grain growth kinetics are described by $d^{1/n}=kt$. The *k* value can be expressed as an Arrhenius equation with the activation energy for grain growth *Q_G_*. The *Q_G_* value of the FeCoNiCrPd HEA reaches 831.9 kJ/mol, and those of the other H/MEAs fall within the range of 300–500 kJ/mol; all are much higher than those of conventional alloys. It is demonstrated that a higher diffusion coefficient *D* value of an alloy leads to a lower *Q_G_* value of the alloy. These characteristics suggest that the lattice distortion effect exhibited in H/MEAs is significant in slowing down the grain boundary motion. Due to the diffusion rate of Mn being the highest among the constituent elements, H/MEAs with Mn have lower *Q_G_* values than their counterparts without Mn due to the addition of Mn or the substitution of Mn for Cr. The lattice distortion δ, which could suppress grain boundary migration, is also correlated to the *Q_G_* value.

## Figures and Tables

**Table 1:** Grain size *d* and hardness *H* of all high/medium entropy alloys recrystallized at different annealing temperatures for 1 h

Alloy	Annealing Temperature *T* (°C) for 1 h	Grain Size *d* (μm)	Hardness *H* (Hv)	Alloy	Annealing Temperature *T* (°C) for 1 h	Grain Size *d* (μm)	Hardness *H* (Hv)
FeCoNiCrMn	800	3.7 ± 0.9	176.6 ± 2.6	FeCoNiMn	800	9.8 ± 2.3	144.5 ± 1.2
	900	13.9 ± 3.6	147.9 ± 2.2		900	27.5 ± 3.1	134.4 ± 1.9
	1000	63.3 ± 9.6	136.1 ± 4.2		950	55.9 ± 10.5	127.6 ± 1.8
	1100	120.2 ± 22.0	132.8 ± 2.8		1000	87.0 ± 8.4	121.9 ± 2.4
	1200	209.6 ± 41.2	128.7 ± 2.4		1100	208.3 ± 27.2	118.9 ± 2.0
FeCoNiCrPd	900	6.9 ± 1.0	225.5 ± 0.9	CoNiCr	800	4.0 ± 0.5	255.1 ± 4.3
	950	29.2 ± 5.2	208.4 ± 3.4		900	12.3 ± 3.3	195.5 ± 3.3
	1000	47.1 ± 9.4	205.6 ± 2.1		1000	69.3 ± 6.0	158.0 ± 4.9
	1100	108.9 ± 12.8	201.6 ± 1.0		1100	101.7 ± 17.4	153.3 ± 3.0
	1200	292.7 ± 31.3	197.7 ± 3.5		1200	152.6 ± 19.2	151.5 ± 5.0
FeCoNiCr	800	4.2 ± 0.8	185.6 ± 4.0	CoNiMn	800	17.2 ± 2.4	157.8 ± 2.6
	900	13.1 ± 3.3	154.9 ± 3.3		850	23.8 ± 2.6	150.9 ± 1.9
	1000	64.2 ± 8.6	133.6 ± 2.5		900	41.4 ± 7.3	145.1 ± 1.5
	1100	108.1 ± 14.8	129.1 ± 7.1		950	83.9 ± 13.7	139.8 ± 2.1
	1200	153.9 ± 16.8	125.4 ± 3.7		1000	166.4 ± 18.6	136.5 ± 1.6

**Table 2:** The *H*_0_, *K*_H_, lattice distortion δ and shear modulus of all high/medium entropy alloys recrystallized at different annealing temperatures for 1 h.

Alloy	*H*_0_ (Hv)	*K*_H_ (Hv·μm^0.5^)	δ × 100	Shear Modulus (GPa) [18]
FeCoNiCrMn	122.3	103.1	1.12	80
FeCoNiCrPd	193.0	85.2	3.66	-
FeCoNiCr	114.7	145.5	1.18	84
FeCoNiMn	112.4	104.1	0.89	77
CoNiCr	128.7	248.7	1.35	87
CoNiMn	126.0	126.8	0.99	77
FeCoNiCrAl_0.3_ [23]	111	227	3.64	-
FeCoNiCrMn [12]	125	69	1.12	-

**Table 3:** Atomic size and Young’s modulus of each constituent element

Element	Effective Radius (pm) [47]	Metallic Radius (pm) [44]	Young’s Modulus (GPa) [45,46]
Fe	126.81	126	211
Co	124.46	125	209
Ni	123.28	124	200
Cr	129.25	128	279
Mn	127.52	127	198
Pd	-	137	121
Al	-	143	70

**Table 4:** The grain growth exponent *n* and kinetic constant *k* of all high/medium entropy alloys recrystallized at 900 °C for different annealing times. The solute drag factor *C_SD_* and corresponding parameters of the solute concentration *C* and diffusivity *D*_0_ for each alloy are also listed.

Alloy	*n*	*k*
FeCoNiCrMn	0.4264	549.3
FeCoNiCrPd	0.2959	727.8
FeCoNiCr	0.3694	1170.9
FeCoNiMn	0.3602	10942.3
CoNiCr	0.3493	1398.4
CoNiMn	0.3559	34626.5

**Table 5:** Activation energy *Q_G_* of all high/medium entropy alloys (H/MEAs) and some conventional alloys. The diffusion coefficient *D*, and corresponding parameters of diffusivity *D*_0_, activation energy for diffusion *Q_D_* and melting temperature *T_m_* for all H/MEAs. The lattice distortion δ of all H/MEAs are listed for showing the correlation between δ and *D*.

Alloy	*Q_G_* (kJ/mol)	*D*_0_ [25] (10^−4^ m^2^/s)	*Q_D_* [25] (kJ/mol)	*T_m_* [25] (K)	*D* (10^−4^ m^2^/s)	δ× 100
FeCoNiCrMn	420.9	9.5	308	1553	9.3	1.12
FeCoNiCrPd	831.9	0.5	258	1560	0.5	3.66
FeCoNiCr	434.3	4.9	309	1695	4.8	1.18
FeCoNiMn	332.5	*D*_Mn_ > *D*_Cr_ ~ *D*_Fe_ > *D*_Ni_ ~ *D*_Co_ [59,60]			>9.3	0.89
CoNiCr	478.8	3.5	330	1690	3.4	1.35
CoNiMn	325.1	*D*_Mn_ > *D*_Cr_ ~ *D*_Fe_ > *D*_Ni_ ~ *D*_Co_ [59,60]			>9.3	0.99
304LN stainless steel [57]	150	-			-	-
AZ31 Mg alloy [58]	110	-			-	-
α-Ti-0.2Pd Ti alloy [56]	133	-			-	-
β-Ti-0.2Pd Ti alloy [56]	56	-			-	-
FeCoNiCrMn [12]	321.7	-			-	-
